# Perpendicular Magnetic Anisotropy in FePt Patterned Media Employing a CrV Seed Layer

**DOI:** 10.1007/s11671-010-9755-2

**Published:** 2010-08-26

**Authors:** Hyunsu Kim, Jin-Seo Noh, Jong Wook Roh, Dong Won Chun, Sungman Kim, Sang Hyun Jung, Ho Kwan Kang, Won Yong Jeong, Wooyoung Lee

**Affiliations:** 1 Department of Materials Science and Engineering, Yonsei University, Seoul 120-749, Korea; 2 Korea Institute of Science and Technology (KIST), Seongbuk-gu, Seoul 136-761, Korea; 3 Nano Process Division, Korea Advanced Nano Fab. Center, Gyeonggi 443-270, Korea

**Keywords:** FePt, CrV underlayer, Patterned media, E-beam lithography

## Abstract

A thin FePt film was deposited onto a CrV seed layer at 400°C and showed a high coercivity (~3,400 Oe) and high magnetization (900–1,000 emu/cm^3^) characteristic of *L*1_0_ phase. However, the magnetic properties of patterned media fabricated from the film stack were degraded due to the Ar-ion bombardment. We employed a deposition-last process, in which FePt film deposited at room temperature underwent lift-off and post-annealing processes, to avoid the exposure of FePt to Ar plasma. A patterned medium with 100-nm nano-columns showed an out-of-plane coercivity fivefold larger than its in-plane counterpart and a remanent magnetization comparable to saturation magnetization in the out-of-plane direction, indicating a high perpendicular anisotropy. These results demonstrate the high perpendicular anisotropy in FePt patterned media using a Cr-based compound seed layer for the first time and suggest that ultra-high-density magnetic recording media can be achieved using this optimized top-down approach.

## Introduction

Conventional planar magnetic recording methods have been facing difficulties in reducing the thickness of a magnetic film and the average grain size in it, which is required for the high bit density [1,2]. Furthermore, these methods showed a bit density limit of about 100 Gbit/in^2^ due to the magnetic moment instability termed 'superparamagnetism' in very small grains and the magnetic exchange interaction between adjacent grains [1-4]. To overcome this limit, a perpendicular magnetic recording was introduced, where magnetic moments are aligned perpendicular to the film plane [5,6]. However, a bit loss still occurs by exchange interaction between neighboring grains. Patterned magnetic media have emerged as a means to prevent this intergranular exchange interaction, thus to achieve the ultra-high density of magnetic recording. To realize the very fine patterned media, a proper material stack and well-optimized fabrication process should be chosen to retain the magnetization in the perpendicular direction with a high perpendicular anisotropy (*K*_*u*_).

FePt is a magnetic material that has been intensively investigated due to its high coercivity (*H*_*c*_ = 1–10 kOe) [7-11] and high magnetocrystalline anisotropy (*K*_*c*_ = 7.0 × 10^7^ erg/cm^3^) [8,10,12]. This material undergoes a transition from chemically disordered face-centered cubic phase (FCC, *A*1 phase) to ordered face-centered tetragonal phase (FCT, *L*1_0_ phase) at a specific temperature, and the transition temperature and perpendicular anisotropy are known to depend on the buffer layer and process employed. A variety of buffer layers have been introduced on Si or glass substrates to grow high quality FCT structures at low temperatures, including Pt, Au, Ag, Ti, and MgO [13-16]. Although *L*1_0_ FePt films on these buffer layers demonstrated an increase in coercivity with respect to the buffer-free films, the ratio of out-of-plane to in-plane coercivities has generally been smaller than 3. Other than these rather conventional buffer layers, Cr-based compounds such as CrW [17] and CrRu [18] have also been examined as underlayers since (200) planes of a body-centered cubic (BCC) Cr were likely to stimulate (001) texture formation of the FCT FePt and to facilitate the FCC-to-FCT transition in FePt layer by forcing the tensile stress to *a*_0_ side of the original FCC FePt [17,18], achieving the ratio of out-of-plane to in-plane coercivities larger than 5 at a relatively low temperature (400°C) [18]. To our knowledge, however, no works have successfully demonstrated the high perpendicular anisotropy in FePt fine-patterned media employing a Cr compound, presumably due to the difficulty in optimal process design.

In this work, we fabricated magnetic recording media by a combination of E-beam lithography and either dry etching (deposition-first process) or lift-off (deposition-last process), where magnetic nano-columns were regularly arranged with a fixed spacing. The magnetic properties and crystal structures were investigated at important steps of the fabrication of the patterned media. The high perpendicular anisotropy is demonstrated in the fine-patterned media, suggesting the feasibility of achieving the ultra-high-density recording media through a well-designed fabrication process.

## Experimental

**Figure 1:**
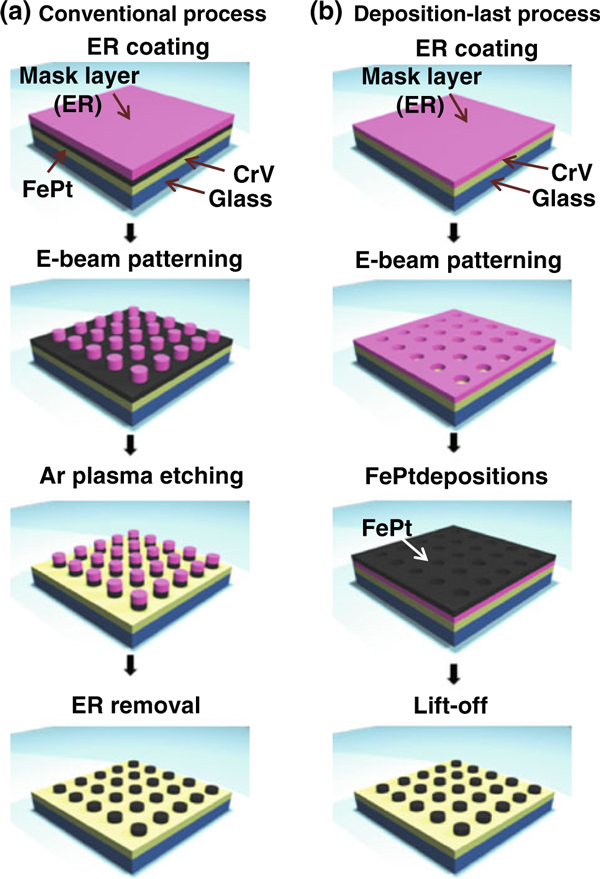
***Schematic pictures* showing fabrication procedures of FePt patterned media: a conventional top-down process and b deposition-last process**.

A 70-nm-thick CrV seed layer was sputter-deposited at 400°C on a glass substrate. Then, a FePt layer 7 nm in thickness was deposited on top of the CrV at 400°C by ultra-high vacuum (UHV, 3 × 10^-8^ Torr) sputtering [19]. Patterned media were fabricated from this film stack, following the conventional top-down process (deposition-first process) shown in Figure 1a. In this process, a type of negative E-beam resist (ER), hydrogen silsesquioxane (HSQ), was used for E-beam lithography. The coated ER was baked at 110°C for 60 s before E-beam irradiation. Going through E-beam exposure and development in tetramethylammonium hydroxide (TMAH), regular ER columns were patterned: typical diameter and pitch of the ER patterns were 100 and 200 nm, respectively. Using the ER patterns as etch masks, the inductively coupled plasma (ICP) Ar etching was performed for 1 min under 15 sccm of Ar flow to transfer the ER patterns onto the film stack. The etching was stopped right below FePt/CrV interface. The ER was finally removed, leaving behind FePt patterns, as shown in the last panel of Figure 1a.

As an alternative process, a lift-off process (deposition-last process) was employed to fabricate the patterned media, as shown in Figure 1b. For this process, a type of positive ER was coated on CrV layer and patterned undergoing E-beam exposure and development steps, leaving behind a regular array of holes of a fixed size (typically, 100 nm). Then, a 7-nm-thick FePt layer was deposited by sputtering at room temperature, followed by a lift-off. The final FePt patterns (the last panel of Figure 1b) were subsequently annealed at 400°C for 1 h to induce a phase transformation from *A*1 to *L*1_0_ phase.

To analyze the crystal structures of as-grown films and patterned media, conventional θ–2θ X-ray diffraction (XRD) was performed using Cu *K*α radiation. Magnetic properties were investigated at room temperature, using a superconducting quantum interference device (SQUID) with a sensitivity of 1 × 10^-6^ emu. Microstructures of the film stacks and top-views of the fabricated patterned media were observed using transmission electron microscopy (TEM) and scanning electron microscopy (SEM), respectively.

## Results and Discussion

**Figure 2:**
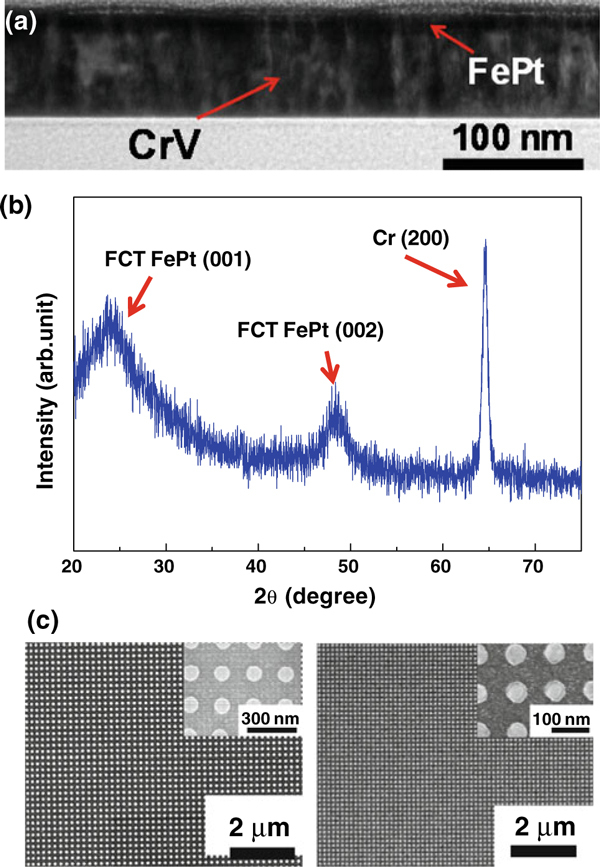
**a Transmission electron microscopy (TEM) image of thin FePt layer on columnar CrV seed layer**. **b** XRD pattern of the as-grown film stack. **c** Scanning electron microscopy (SEM) images of FePt patterns of 100 nm diameter (left) and 50 nm diameter (right), respectively. *Insets* show magnified views of the respective patterns for clarity.

Figure 2a shows a TEM image of an as-grown FePt/CrV film stack. The CrV seed layer exhibits a well-developed columnar grain structure. From our previous study, the well-defined columnar grains of the CrV layer was found to induce perpendicularly oriented grains in a thin FePt overlayer, which resulted in *L*1_0_ FePt film at a moderate temperature [20]. To confirm this, we performed a XRD measurement on the as-grown FePt/CrV film stack. As seen in Figure 2b, characteristic FCT (001) and (002) peaks are observed without any FCC peaks, indicating that the FePt film is really in the *L*1_0_ phase. The noisy baseline and rather broad FePt peaks are probably due to the very small thickness (7 nm) of the FePt film. Using this *L*1_0_ FePt film on a CrV seed layer, FePt patterned media were fabricated. Figure 2c shows the FePt patterns of different sizes (100 and 50 nm in diameter) fabricated by the combined use of E-beam lithography and Ar plasma etching. The FePt nano-columns having a circular cross section are regularly arrayed on the CrV/glass substrates. The spacing between neighboring nano-columns is the same as its diameter, making the pitch a twofold of the diameter (200 and 100 nm for the respective pattern). From the figure, it is apparent that FePt patterns down to 50 nm in size (100 nm in pitch) can be fabricated by our top-down approach. As a matter of fact, we confirmed that the pattern size could be reduced to 25 nm with 50 nm pitch. Below this size limit (25 nm), the nano-columns started to be deformed, leading to a partly connected array.

**Figure 3:**
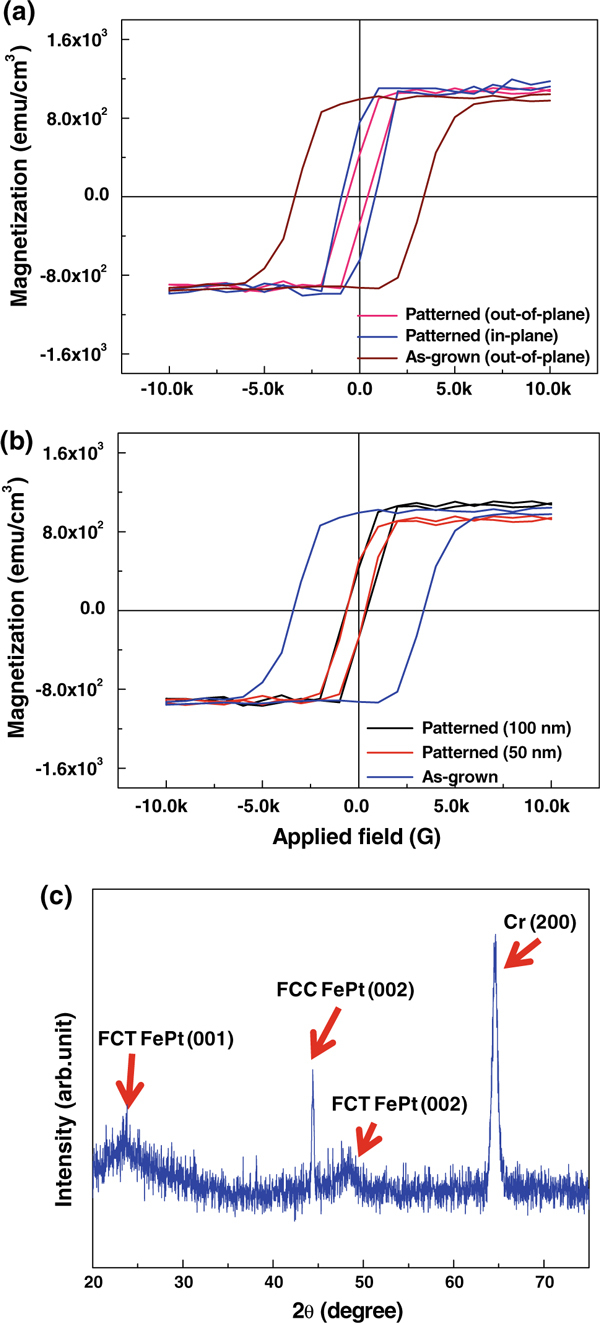
**a *M* vs. *H* curves at room temperature for the as-grown film (out-of-plane) and a patterned medium with 100-nm-sized columns (out-of-plane and in-plane)**. **b** Out-of-plane *M* vs. *H* curves for the as-grown film and patterned media with 100 and 50 nm columns. **c** XRD pattern of a patterned medium with 100-nm-sized columns.

We carried out magnetic field sweepings on the patterned media to investigate the magnetization (*M*) versus magnetic field (*H*) behaviors of the media, using a SQUID. Figure 3a shows the *M* versus *H* loops measured at room temperature for the as-grown FePt film (out-of-plane) and a patterned medium with 100-nm-sized columns (both in-plane and out-of-plane). The saturation magnetization (*M*_s,film_) and coercivity (*H*_c,film_) of the as-grown film are 900–1,000 emu/cm^3^ and ~3,400 Oe, respectively, which are close to those previously reported for FePt *L*1_0_ phase [8]. These values and the high ratio of remanent magnetization (*M*_r,film_) to saturation magnetization, *M*_r,film_/*M*_s,film_ ≈ 1, may be another indicators that the FePt film was ordered into *L*1_0_ phase during deposition at 400°C. It is believed that the formation of complete *L*1_0_ phase at a temperature lower than widely adopted post-annealing temperatures (500–800°C) [15,21-23] is attributed to both the high surface diffusivity of adatoms at the elevated deposition temperature and good morphology transfer from the CrV seed layer to a growing FePt film.

However, the coercivities (*H*_c,pattern_ = 450–900 Oe) of the patterned medium appear to be 4 to sevenfold smaller than *H*_c,film_ both in film plane and normal to plane, although its saturation magnetizations (*M*_s,pattern_) are similar to *M*_s,film_. In addition, the ratio (*M*_r,pattern_/*M*_s,pattern_ = 0.4–0.7) of *M*_r,pattern_ to *M*_s,pattern_ for the medium is smaller than that of the as-grown film. Recollecting that the coercivity and *M*_r_/*M*_s_ ratio are more structure-sensitive than the saturation magnetization, these results suggest that the chemically ordered FCT structure was destroyed and replaced by the chemically disordered FCC structure at least partially during ICP Ar etching. To verify this presumption, we carried out the XRD analysis on the patterned medium. Indeed, it is shown from Figure 3c that the FCT (002) peak is weak and instead, a FCC (002) peak is clearly developed around 2θ = 44.5°, justifying the propriety of the above presumption. We think that the large decrease in coercivity for the patterned medium originated from the relaxation of magnetocrystalline anisotropy (*K*_*c*_) due to the chemical disordering in the FePt patterns [7,24,25]. This is because shape anisotropy (*K*_*d*_ α α$M_{s}^{2}$, where α is the demagnetization factor) strengthens the perpendicular alignment of magnetic moments, and magnetoelastic anisotropy (*K* where is the magnetostriction constant and is the stress in film) remains almost unchanged via patterning [26]. The Ar-ion penetration into the FePt film and a large momentum delivered from impinging Ar ions may be primary sources for the collapse of the FCT structure. The drastic decrease in coercivity was also observed in other patterned media with different pattern size, as shown in Figure 3b. It is seen from this figure that both coercivities and *M*_r_/*M*_s_ ratios for patterned media are significantly reduced from the values of the as-grown film irrespective of pattern size, reflecting the FCT structure was collapsed for samples undergoing Ar plasma etching as confirmed by the XRD result in Figure 3c.

**Figure 4:**
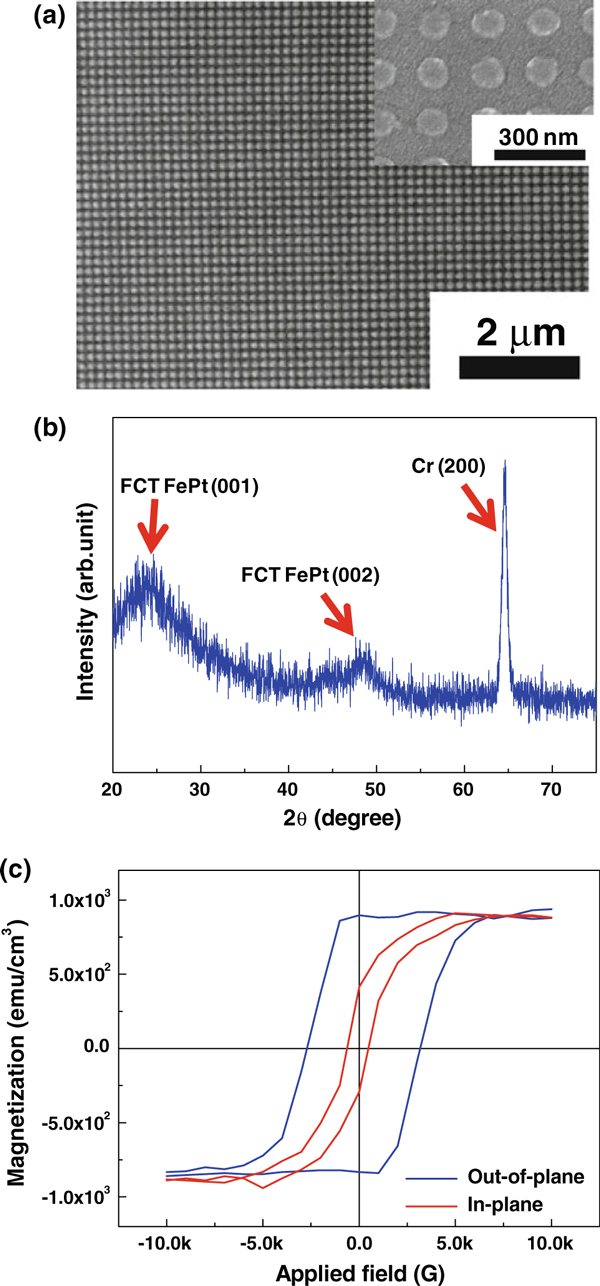
**a SEM image and b XRD pattern of FePt patterns of 100 nm diameter fabricated by the deposition-last process**. The *inset* in **a** shows a magnified view of the pattern for clarity. **c** Comparison of *M* vs. *H* curves for the patterned medium in out-of-plane and in-plane directions.

To avoid this direct exposure of FePt film to Ar plasma, we modified the fabrication procedure of patterned media as illustrated in Figure 1b. Based on this deposition-last process, the FePt film remains intact because no ion impingement is involved in whole fabrication steps. Figure 4a shows the FePt patterns produced by a combination of E-beam lithography and FePt lift-off. The FePt patterns of 100 nm size (200 nm pitch) are circular in shape and uniformly spaced from their neighbors, making pattern quality comparable to that of the top-down patterns mentioned above (see Figure 2c for comparison). A XRD measurement on the deposition-last patterned medium confirms that this modified process allows for realization of the *L*1_0_ phase in fine-patterned FePt, as seen from Figure 4b. Magnetic hysteresis loops for this deposition-last patterned medium are shown in Figure 4c for both applied field directions of out-of-plane and in-plane. Now, a perpendicular anisotropy is clearly observed, making the direction perpendicular to film plane a magnetic easy axis. The coercivities in out-of-plane and in-plane directions are approximately 3,000 and 600 Oe, respectively, resulting in *H*_c,out_/*H*_c,in_ ≈ 5 for this patterned medium. The strong perpendicular magnetic anisotropy is also supported by the perfect squareness (*M*_r,out_/*M*_s,out_ ≈ 1) of *M*-*H* curve in the out-of-plane direction, while this ratio falls to a half (*M*_r,in_/*M*_s,in_ = 0.52) in film plane.

Comparing the out-of-plane coercivity of this patterned medium with that of the as-grown film prepared by the deposition-first process, there exists a small difference of about 400 Oe. We believe that this magnitude of difference is reasonable since the surface migration of adatoms during film growth at elevated temperature (400°C) is easier compared to solid-state diffusion of constituents during post-annealing at the same temperature. Qiu et al. also fabricated FePt patterned media with underlayers such as Ag and MgO, employing a similar deposition-last process [15]. In their media, however, the FCC-to-FCT phase transition was retarded to higher temperatures and no perpendicular anisotropy was observed. Assuming that the magnetocrystalline anisotropy is a primary source of our perpendicular anisotropy as explained above, the perpendicular anisotropy is proportional to the coercivity and saturation magnetization in the out-of-plane direction, *K*_*u*_*H*_c,out_*M*_s,out_. In our 100-nm-sized FePt patterns fabricated by the deposition-last process, the values are measured to be 3,000 Oe and 870 emu/cm^3^, respectively. These values are comparable to those of the previously reported FePt thin films on other Cr-based compounds such as CrW [17] and CrRu [18], demonstrating the high efficiency of the CrV seed layer in fabricating patterned media with a high perpendicular anisotropy. Besides this, our results disclose important implications: (1) a root cause of the magnetic property degradation of FePt patterned media fabricated by a conventional deposition-first process is chemical disordering incurred by ion plasma etching. (2) The deposition-last process is desirable for implementing ultra-high-density patterned media, and the post-annealing temperature can be maintained low by the support of an appropriate seed layer.

## Conclusions

We fabricated FePt-based perpendicular patterned media using a selective combination of E-beam lithography and either Ar plasma etching (deposition-first process) or FePt lift-off (deposition-last process). A FePt film on a CrV seed layer grown at 400°C showed a high perpendicular anisotropy indicating *L*1_0_ phase of FCT structure formed during deposition, whereas the anisotropy was collapsed in patterned media fabricated from the film stack. We employed the deposition-last process to avoid chemical and structural disordering by impinging Ar ions. For a patterned medium with 100 nm patterns made by this process, the out-of-plane coercivity was measured to be fivefold larger than its in-plane value and the out-of-plane *M*-*H* curve exhibited a perfect squareness, indicating a high perpendicular anisotropy. To our knowledge, this is the first demonstration of a high perpendicular anisotropy in patterned media using a Cr-based compound seed layer. Furthermore, the deposition-last process may be a promising way to achieve ultra-high-density patterned media due to its maintainability of perpendicular anisotropy and controllability of pattern size and shape.
